# Essential genes *Ptgs2*, *Tlr4*, and *Ccr2* regulate neuro-inflammation during the acute phase of cerebral ischemic in mice

**DOI:** 10.1038/s41598-023-40255-w

**Published:** 2023-08-10

**Authors:** Hongxiang Jiang, Zhiqiang Sun, Xiwei Zhu, Fei Li, Qianxue Chen

**Affiliations:** 1 Department of Neurosurgery, Renmin Hospital of Wuhan University, 238 Jiefang Road, Wuchang Distict, Wuhan, 430060 Hubei Province China; 2 Central Laboratory, Renmin Hospital of Wuhan University, Wuhan, 430060 Hubei Province China

**Keywords:** Immunogenetics, Immunology, Biomarkers, Neurology, Stroke

## Abstract

Ischemic stroke (IS) is associated with changes in gene expression patterns in the ischemic penumbra and extensive neurovascular inflammation. However, the key molecules related to the inflammatory response in the acute phase of IS remain unclear. To address this knowledge gap, conducted a study using Gene Set Enrichment Analysis (GSEA) on two gene expression profiles, GSE58720 and GSE202659, downloaded from the GEO database. We screened differentially expressed genes (DEGs) using GEO2R and analyzed 170 differentially expressed intersection genes for Kyoto Encyclopedia of Genes and Genomes (KEGG) pathway enrichment and Gene Ontology (GO) analysis. We also used Metascape, DAVID, STRING, Cytoscape, and TargetScan to identify candidate miRNAs and genes. The targeted genes and miRNA molecule were clarified using the mice middle cerebral artery occlusion-reperfusion (MCAO/R) model. Our findings revealed that 170 genes were correlated with cytokine production and inflammatory cell activation, as determined by GO and KEGG analyses. Cluster analysis identified 11 hub genes highly associated with neuroinflammation: *Ccl7*, *Tnf*, *Ccl4*, *Timp1*, *Ccl3*, *Ccr1*, *Sele*, *Ccr2*, *Tlr4*, *Ptgs2*, and *Il6*. TargetScan results suggested that *Ptgs2*, *Tlr4,* and *Ccr2* might be regulated by *miR-202-3p*. In the MCAO/R model, the level of *miR-202-3p* decreased, while the levels of *Ptgs2*, *Tlr4,* and *Ccr2* increased compared to the sham group. Knockdown of *miR-202-3p* exacerbated ischemic reperfusion injury (IRI) through neuroinflammation both in vivo and in vitro. Our study also demonstrated that mRNA and protein levels of *Ptgs2*, *Tlr4,* and *Ccr2* increased in the MCAO/R model with *miR-202-3p* knockdown. These findings suggest that differentially expressed genes, including *Ptgs2*, *Tlr4,* and *Ccr2* may play crucial roles in the neuroinflammation of IS, and their expression may be negatively regulated by *miR-202-3p*. Our study provides new insights into the regulation of neuroinflammation in IS.

## Introduction

Ischemic stroke (IS), which is caused by a lack of blood and oxygen supply to the brain^1^, accounts for approximately 85% of stroke casualties. It is a major economic and social burden worldwide^2^. Reperfusion therapy is the only effective treatment for salvaging the penumbra in IS^3^. However, most neuroprotective agents that have shown efficacy in preclinical trials have failed in clinical trials, highlighting the urgent need to seek alternative therapeutic targets for IS^4^. With a better understanding of the mechanisms of IS, it is now known that ischemic cell stress leads to a variety of pathogenic events, including excitotoxicity, inflammation, oxidative stress, and mitochondrial disturbances. These IS-related pathogenic events may amplify the ischemic core and impair post-stroke recovery and the regenerative response of the brain.

It is widely accepted that macrophage and microglia activation mechanisms are involved in the pathology of IS. Therefore, modulating inflammation and neurological recovery are considered promising strategies to improve long-term prognosis after ischemia–reperfusion injury (IRI)^5^. Furthermore, reactive microglia have a dual role in IS, which is directly correlated with the phenotypes of microglia. The phenotypic changes between M1 and M2 in microglia are similar to those in macrophages. Previous studies have suggested that modifying microglial polarization may be a potential treatment for IS^6^. In addition, neuroinflammatory therapies have been shown to have a broader treatment window compared to thrombolytic therapies^7^. To date, the inflammation process after IS has not been fully explained. Therefore, it is important to explore the patterns of genetic alterations in the acute phase of IS to identify more key genes associated with inflammation and gain new targets for intervention.

The complexity of the brain as an organ and the diversity of its cell types have hindered our understanding of the molecular basis of IS. Gene expression regulation plays a vital role in the complex biological phenomena observed in cells. Therefore, new high-throughput sequencing technologies can aid in investigating the molecular basis of the pathological processes in IS, such as neuronal apoptosis, ischemic damage, response to therapy, and inflammatory cell infiltration^8^. However, some deviation may occur due to different experimental models, ischemic time, and even differences in animal species^9^. Furthermore, there are many time-dependent events in the inflammatory response of IS, including astrocyte proliferation, microglial activation^10^, and clearance of dead tissue by macrophages^11^. This cascade of inflammatory events has been found to evolve over time and involves the expression patterns of various molecules. Unfortunately, microRNAs (miRNAs) have been shown to function to play a vital role in the inflammatory cascade during cerebral IRI. Therefore, it is of theoretical importance to construct miRNA-mRNA networks to discover potential molecular therapeutic targets^12^.

Therefore, we screened two mRNA datasets obtained from mice with early acute IS in the GEO database to identify key genes and miRNAs related to the development of inflammatory responses. Furthermore, we performed the stable mice MCAO/R model to validate the identified miRNAs and key genes. The temporal distribution of the three central genes in the present study was characterized to further elucidate the key molecular mechanisms of inflammation in IS, which is expected to provide the basis for effective IRI therapies.

## Results

### DEGs identification

**Figure 1:**
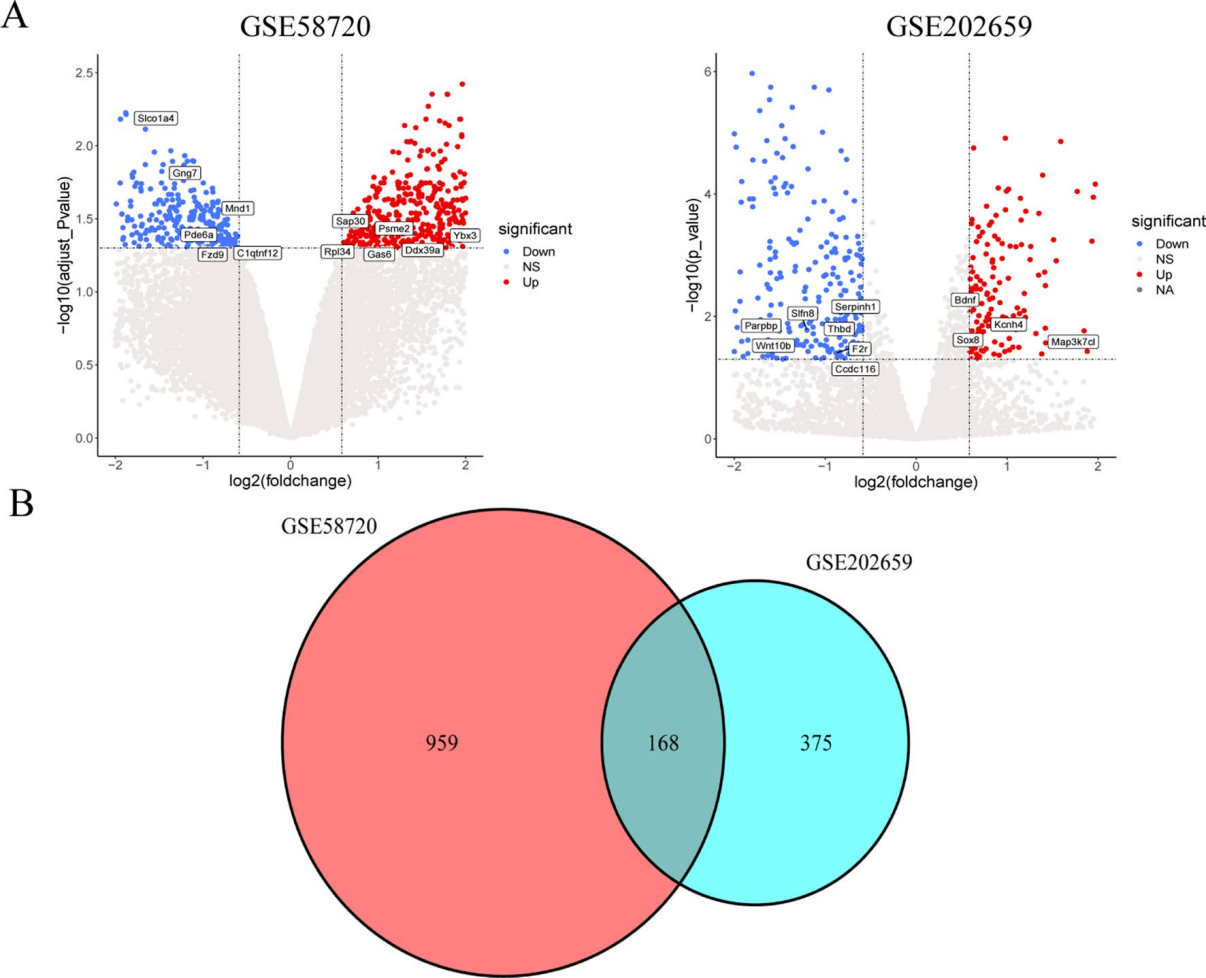
Identification of DEGs in the brain of rats after stroke. (**A**) Volcano plot representing DEGs between MCAO/R groups and SHAM groups shows DEGs in GSE58720 and GSE202659 datasets, respectively. (**B**) Venn diagrams showing the overlaps of numbers of DEGs between 2 selected GEO datasets.

We screened two microarray datasets in the GEO database to identify inflammation-related genes in IS. The GSE58720 dataset includes 3 SHAM samples and 3 MCAO samples, while the GSE202659 dataset includes 3 SHAM samples and 3 MCAO samples. The sequencing information of both datasets comes from mice and belongs to the GPL10787-9758 and GPL24247 platforms, respectively. Using the criteria of adjusted P-value < 0.05 and |logFC| > 0.0585, we identified 1127 DEGs in the GSE58720 data set, and 543 DEGs in the GSE202659 dataset. The volcano plot is depicted in Fig. 1A. We further filtered and mapped these genes using a Venn diagram (Fig. 1B) and found 168 overlapping genes that were differentially expressed in both datasets.

### Gene set enrichment analysis (GSEA)

**Figure 2:**
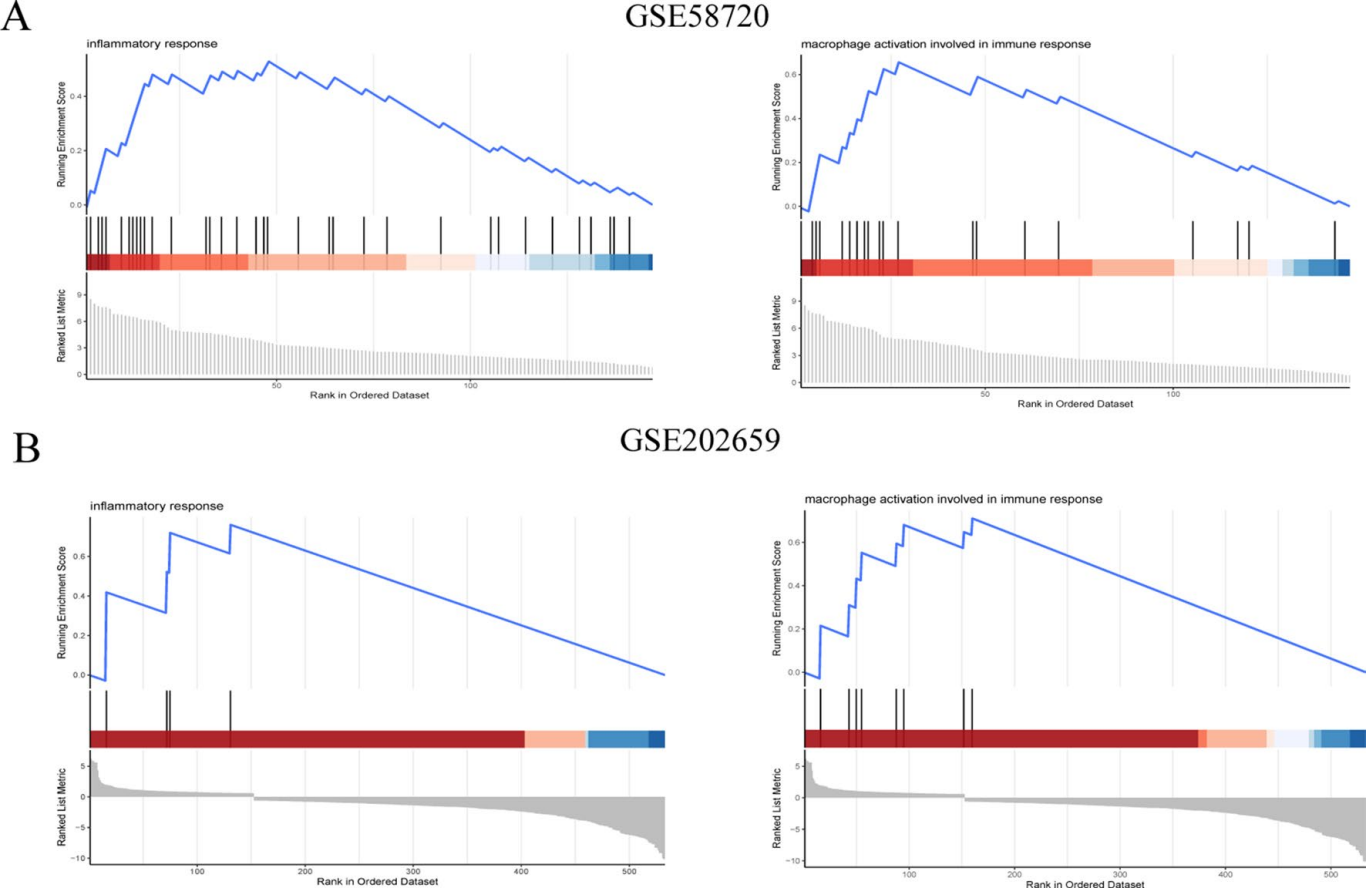
GSEA analysis of the two GEO datasets. (**A**) GSEA analysis results for GSE58720. (**B**) GSEA analysis results for GSE202659.

We performed GSEA analysis using the two selected databases to gain a preliminary understanding of the main roles of all genes. All gene expression information was uploaded to the GSEA package in R, and the C2, C5, and C7 gene set databases were selected to analyze gene expression profiles. The significantly enriched gene sets were determined using a default cut-off q value < 0. 25 and P-value < 0.05. It was found that both gene sets were significantly enriched in coagulation cascades, compliment, and defense response (Fig. 2A,B), which may be related to neurovascular inflammation after IS.

### KEGG and GO enrichment analysis of DEGs

**Figure 3:**
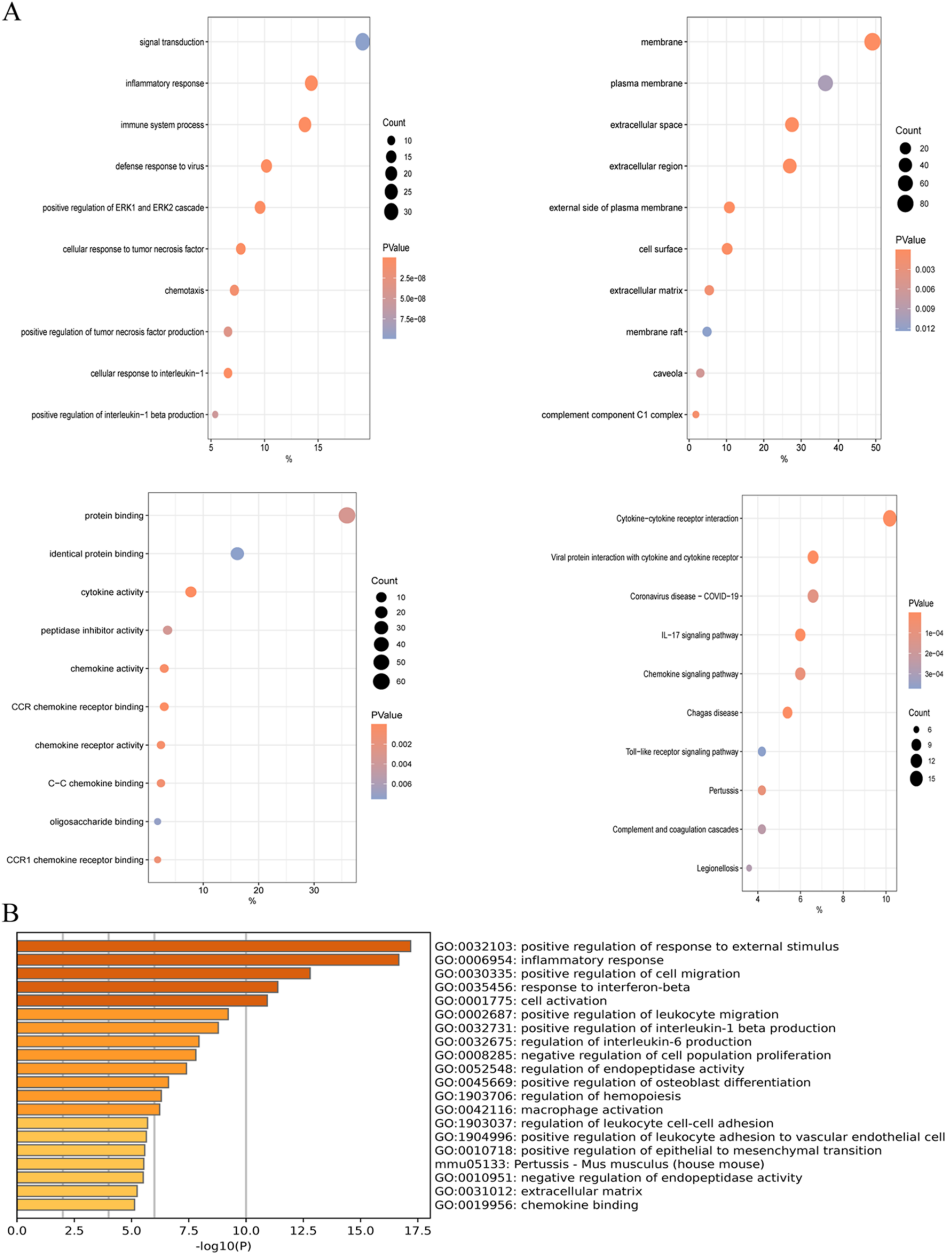
Functional Enrichment Analysis of DEGs. (**A**) Bubble plot of GO and KEGG enrichment analysis results from the DAVID online website^49^. (**B**) Bar graph of GO and KEGG enrichment analysis results from the online website of Metascape.

After identifying the 168 overlapping genes, KEGG and GO enrichment analyses were conducted using Metascape and DAVID online sites. The results from the DAVID database were presented in a bubble plot (Fig. 3A). The biological processes associated with the 168 overlapping genes were found to be significantly enriched in the positive regulation of tumor necrosis factor production and inflammatory response. The cellular components enriched in the analysis were the extracellular region, cell surface, and extracellular space. For molecular function, changes were primarily enriched in chemokine activity and cytokine activity. KEGG analyses indicated that MCMs were associated with the IL-17 signaling pathway and the Toll-like receptor signaling pathway.

The results of the analysis with Metascape are presented in Fig. 3B. The overlapping genes were primarily enriched in the inflammatory response, macrophage activation, and positive regulation of response to external stimulus. The top 20 GO and KEGG enrichment items were classified into 4 functional groups: KEGG pathway (1 item), molecular function (1 item), biological process (17 items), and cellular component (1 item). The 168 overlapping genes were mainly enriched in response to wounding, inflammatory response, immune effector process, regulation of cell adhesion, regulation of cytokine production, response to lipopolysaccharide, angiogenesis, positive regulation of cell death, apoptotic signaling pathway, regulation of hemopoiesis, positive regulation of reactive oxygen species metabolic process, phagocytosis, acute inflammatory response, regeneration, negative regulation of response to external stimulus, response to interleukin-1, and response to extracellular stimulus. The cellular component associated with these genes was the membrane. The molecular function of these genes was integrin binding.

**Figure 4:**
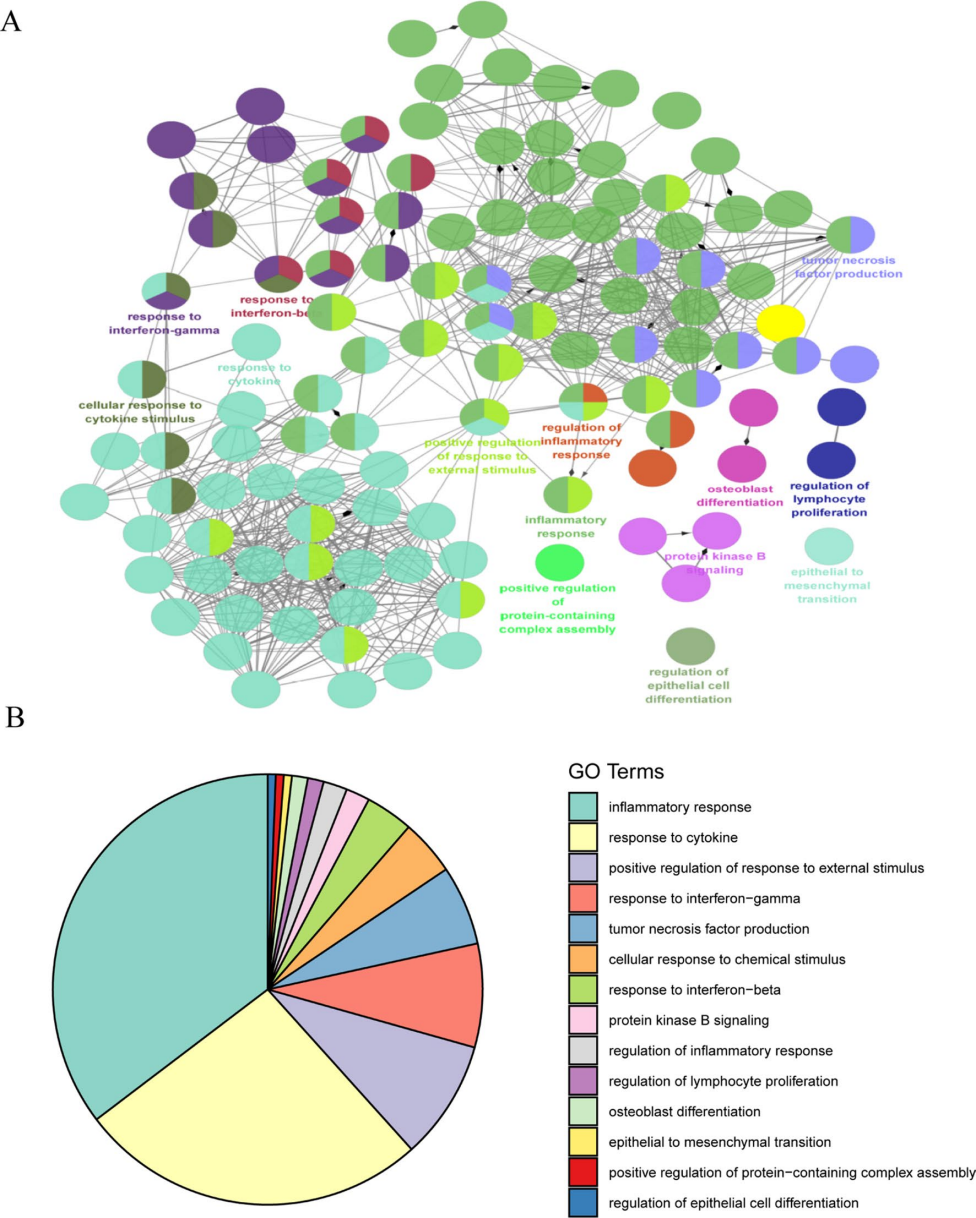
Functional enrichment analysis of DEGs using ClueGO. (**A**) ClueGO was used to analyze the interaction network of enriched biological processes; multiple color dots imply that a DEG is involved in multiple biological processes. (**B**) The pie charts show the enrichment of various biological processes; the gray sector represents paths with a proportion < 1.8%.

Then, we used the ClueGo plug-in of Cytoscape software to visualize the interaction network of biological processes enriched by the 168 genes. The top 14 enriched items are presented as a pie chart (Fig. 4A,B), with the following biological processes being highly enriched: regulation of inflammatory response, cytokine response, positive regulation of response to external stimulus, response to interferon-gamma, tumor necrosis factor production, cellular response to chemical stimulus, response to interferon-beta, protein kinase B signaling, regulation of inflammatory response, regulation of lymphocyte proliferation, osteoblast differentiation, epithelial to mesenchymal transition, positive regulation of protein-containing complex assembly, and regulation of epithelial cell differentiation. Most of these modules are associated with inflammatory processes and inflammatory cell activation.

### Construction of protein–protein interaction (PPI) network for gene clusters

**Figure 5:**
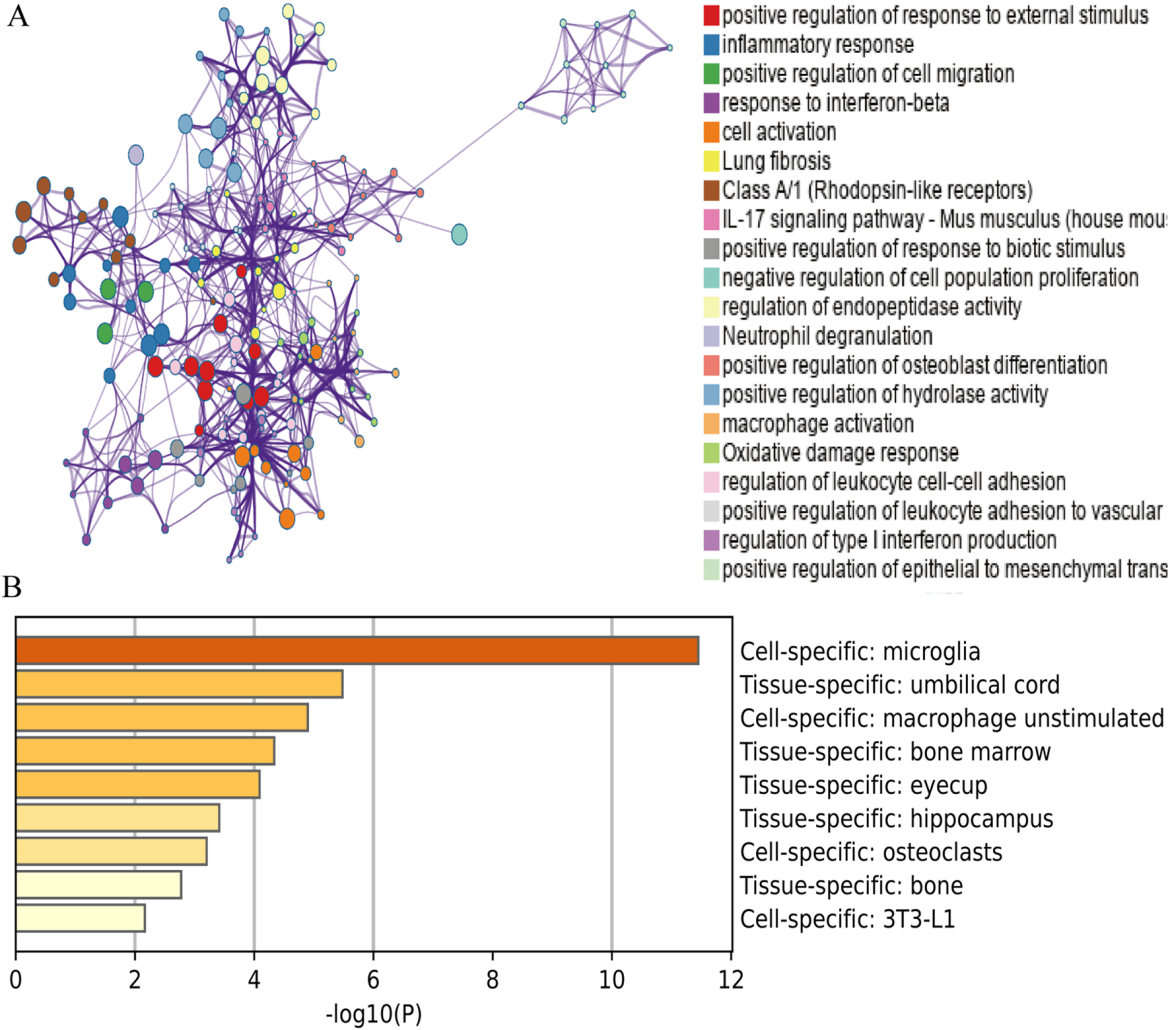
PPI network and function module identification. (**A**) Protein–protein interaction network was processed with Metascape, and different clusters were noted with different colors. (**B**) Major tissue and cell types associated with the DEGs.

To identify key genes from DEGs, we uploaded the 168 intersected genes to Metascape and obtained their relationship with GO items (Fig. 5A,B). Specifically, we found that the red cluster contained genes associated with inflammatory responses. In total, 30 genes were correlated with biological processes related to inflammation. These 30 genes were Bdkrb2, C1qa, C3ar1, C5ar1, Cebpb, Cxcr2, Ccr1, Ccr2, S1pr3, Fut4, Hck, Il6, Acod1, Cd180, Naip2, Ptgs2, S100a9, Ccl11, Ccl3, Ccl4, Ccl7, Sele, Serpina3n, Timp1, Tnf, Trex1, Tlr2, Ccl24, Pycard, and Ticam1. For further analysis, we selected genes related genes to the inflammatory response.

### Screening of inflammatory response related genes

**Figure 6:**
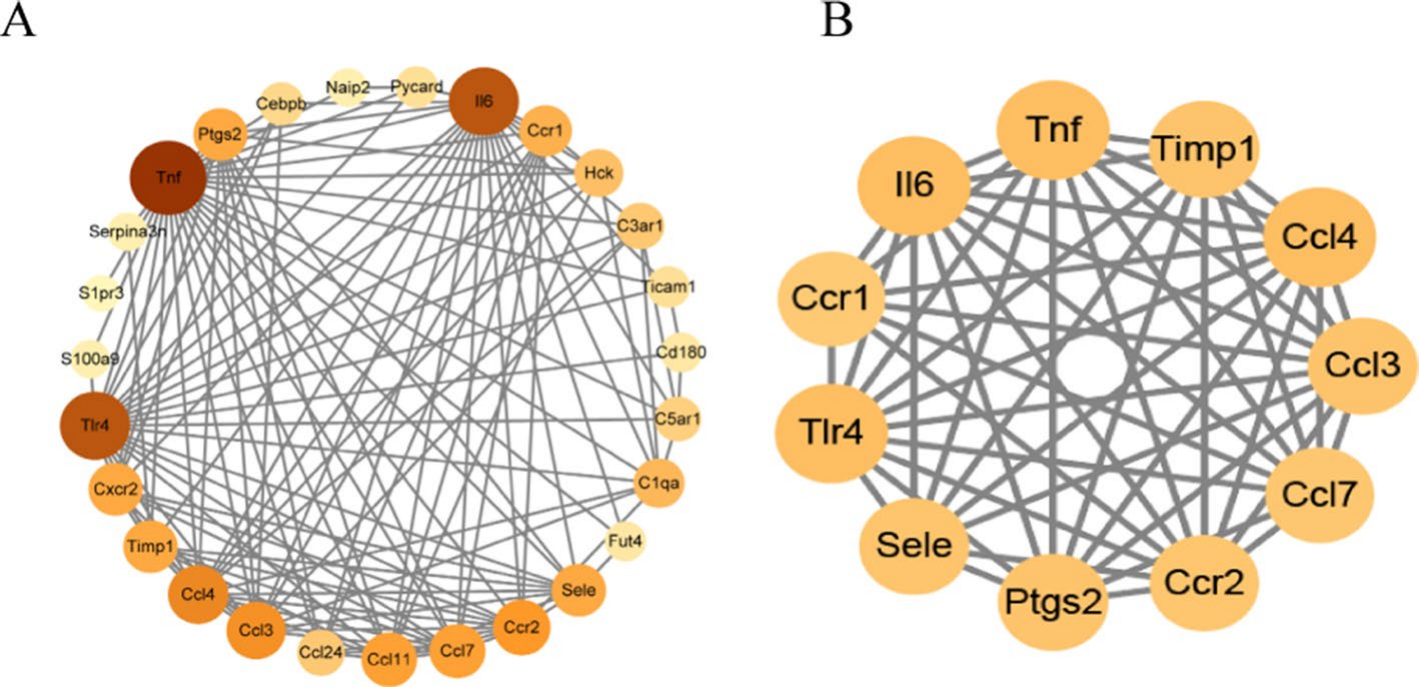
Multi-tools screening for genes highly associated with inflammation. (**A**) Cytoscape software exhibited the correlations, identified by STRING website, between the genes associated with inflammatory response; the size of circles and the depth of colors represented the correlation size. (**B**) The hub genes, associated with inflammatory responses, identified by MCODE function in Cytoscape software.

To screen for genes related to the inflammatory response, we uploaded the 30 highly correlated genes to STRING and Cytoscape (Fig. 6A,B) for further analysis. The protein–protein interaction network constructed in Cytoscape showed the correlation between these 30 genes and their respective intensities, which were represented by the size and color of the bubbles. In Fig. 6B, we identified eleven hub genes that were related to the inflammatory response using the MECODE plugin in Cytoscape. These genes were *Ccl7*, *Tnf*, *Ccl4*, *Timp1*, *Ccl3*, *Ccr1*, *Sele*, *Ccr2*, *Tlr4*, *Ptgs2*, and *Il6*.

### The miRNA mining and interaction network analysis

**Figure 7:**
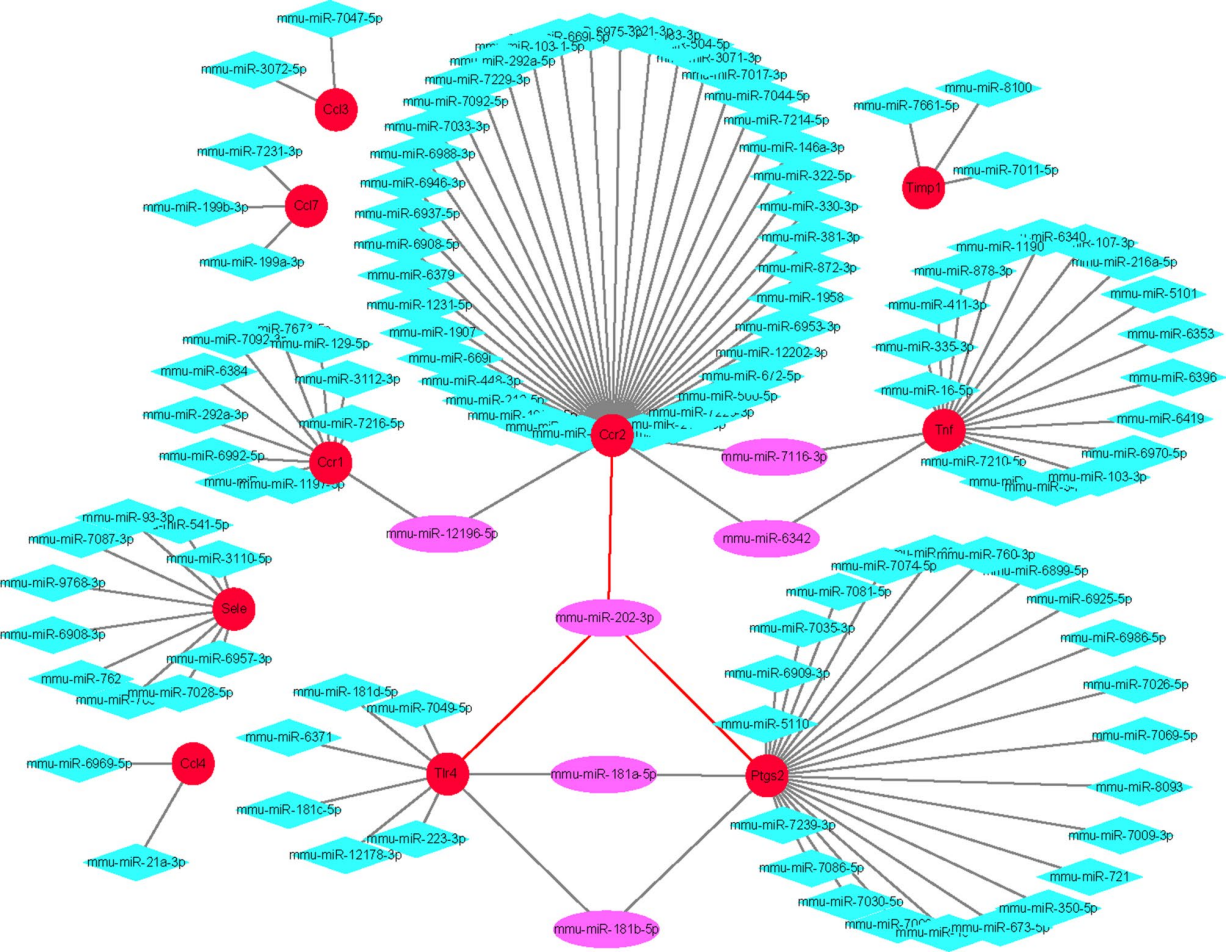
miRNA Prediction with Target Scan. Interaction network between genes involved in the inflammatory response and its targeted miRNAs. Genes are colored in red, and miRNAs are colored in pink, and the red lines means the connections between hub genes and hub miRNA.

In our previous analysis, we identified 11 hub genes that were highly correlated with the inflammatory response: *Ccl7*, *Tnf*, *Ccl4*, *Timp1*, *Ccl3*, *Ccr1*, *Sele*, *Ccr2*, *Tlr4*, *Ptgs2*, and *Il6*. To further investigate the potential regulatory mechanisms of these genes, we conducted a gene-miRNA analysis using the miRWalk website. The selection criteria were based on target gene binding regions in the 3′UTR, seed match > 7 mer-1A, and context +  + score percentile > 90. We used Cytoscape to construct an interaction network (Fig. 7) of the miRNAs that might regulate these genes and clarify their potential roles. Our analysis revealed that *miR-202-3p* regulates three candidate target genes, *Ptgs2*, *Tlr4,* and *Ccr2*, which may be involved in inflammatory response after IS.

### Validation of key miRNAs and genes correlate with inflammation in MCAO/R model

**Figure 8:**
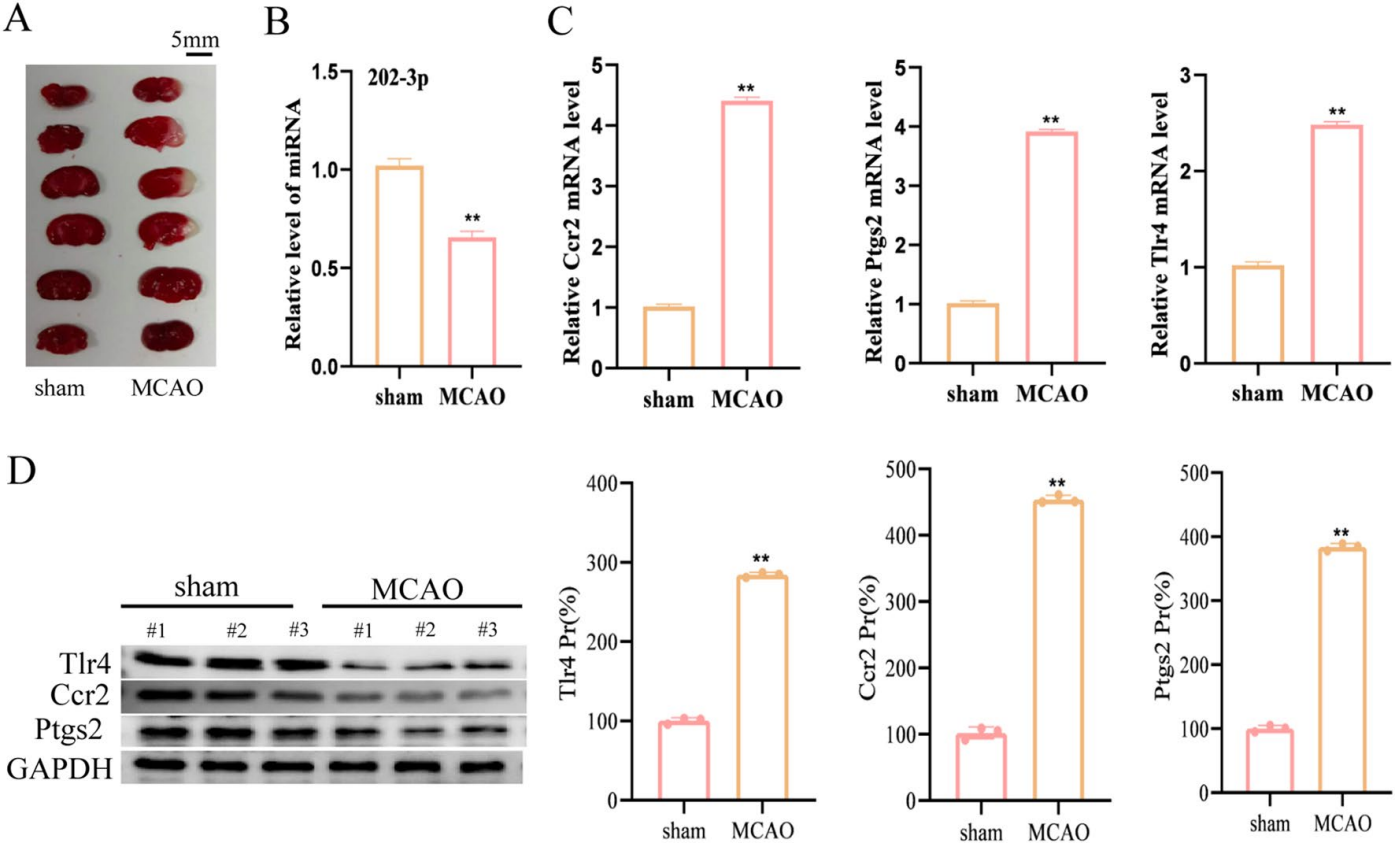
Validation of critical genes and miRNAs in the MCAO model. (**A**) TTC staining of rat MCAO model. (**B**) RT-PCR verification of *miR-202-3p* sham and MCAO group. (**C**) RT-PCR verification of critical genes between sham and MCAO group. (**D**) The protein levels of the targeted genes were determined in the brain tissues from the MCAO group and the sham group. Mean ± SEM, n = 3, **p < 0.01, vs Sham group.

To validate the correlation between *miR-202-3p* and *Ptgs2*/*Tlr4*/*Ccr2*, we divided mice into sham and MCAO/R groups. We obtained brain tissues from the mice MCAO/R model 24 h after ischemia–reperfusion, and confirmed the successful MCAO/R model using TTC staining (Fig. 8A). We detected the levels of miRNAs and candidate genes in the mice ischemic penumbra. The results indicated that the level of *miR-202-3p* decreased at 24 h after IRI, while the expression of *Ptgs2*, *Tlr4,* and *Ccr2* increased (Fig. 8B,C). Additionally, we conducted western blot assays to analyze the protein levels of Ptgs2, Tlr4, and Ccr2 between the sham group and MCAO/R group, which were consistent with the results of the RT-PCR in vivo (Fig. 8D). The above results show that *miR-202-3p* could regulate three post-ischemic upregulated genes: *Ptgs2*, *Tlr4,* and *Ccr2*, which play a vital role in the acute phase of IS.

### The Ptgs2/Tlr4/Ccr2 are regulated by miR-202-3p in inflammation response of IS

**Figure 9:**
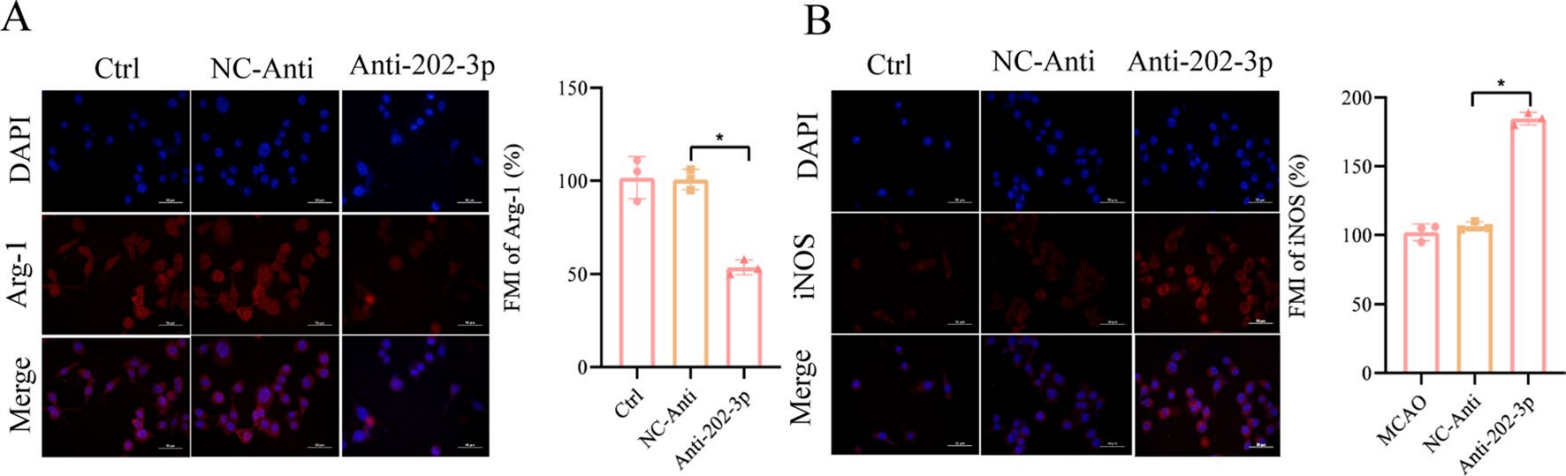
Knockdown of *miR-202-3p* promoted the polarization of microglias to M1 after OGD treatment. (**A**) Representative images of iNOS in microglias. (**B**) Representative images of Arg-1 in microglias. Scale bars = 50 μm.

**Figure 10:**
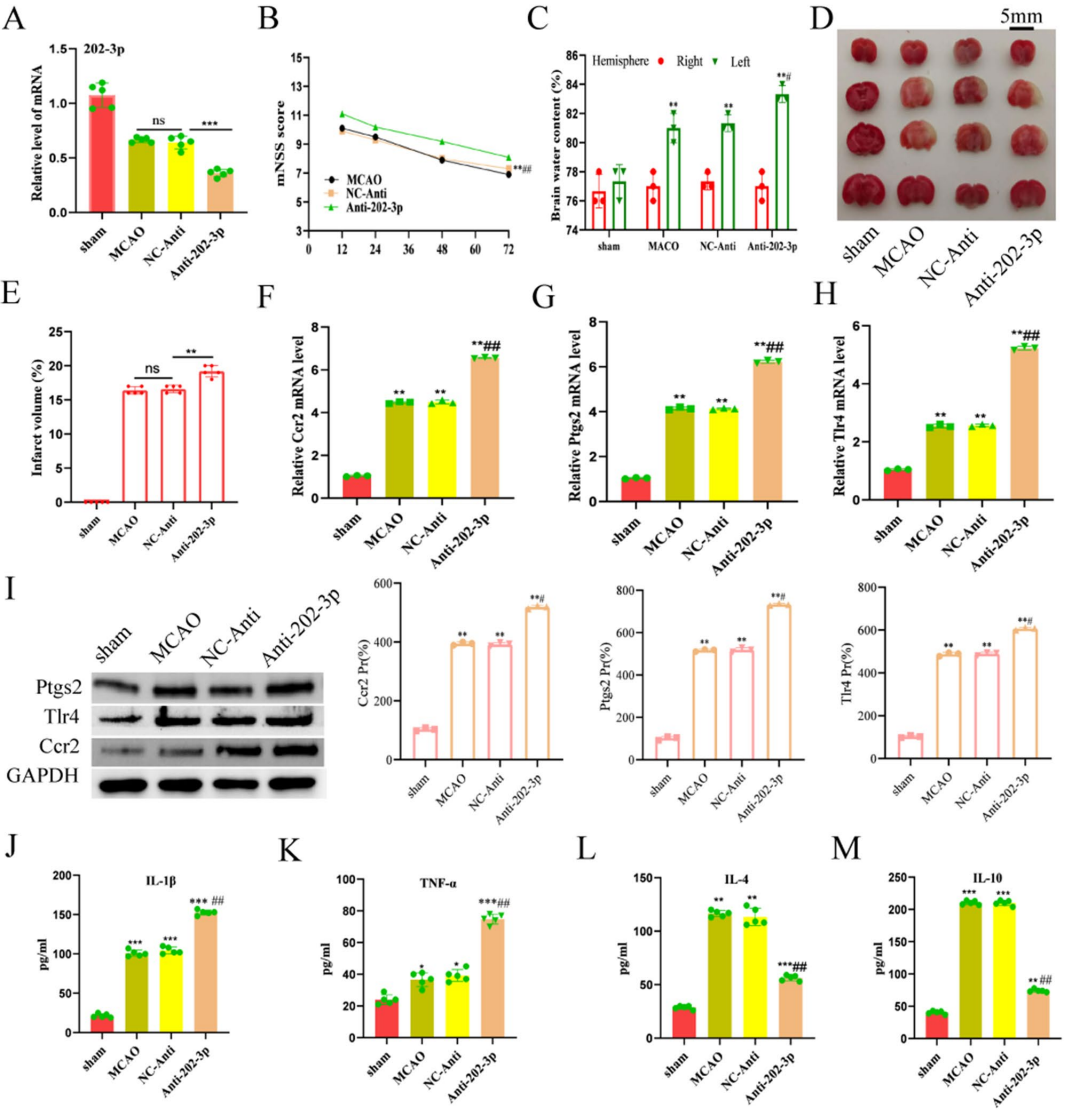
MiR-202-3p knockdown increased the infarct volume and pro-inflammatory cytokines after MCAO/R. (**A**) The expression of *miR-202-3p* was detected with qPCR with different treatments. Data are presented as the mean ± SEM, n = 5, ***p < 0.001 versus NC-Anti group, ns p > 0.05 versus MCAO/R, Mann–Whitney test. (**B**) Time-course of modified neurological severity scores. Data are presented as the mean ± SD, n = 5, **p < 0.01 versus NC-Anti group, ^##^p < 0.01 versus MCAO/R. Mann–Whitney test. (**C**) The brain water content was measured to reflect the severity of brain edema. Bars indicate the mean ± SD, n = 3, **p < 0.01 versus sham group, *p < 0.05 versus sham group, ^#^p < 0.05 versus NC-Anti group, t-test. (**D**) TTC-stained brain slices in sham. (**E**) Comparison of the percentages of infarct volume between each group of mice. n = 5, **p < 0.01 versus NC-Anti group, Mann–Whitney test. (**F**–**H**) The expression of *Ptgs2, Tlr4* and *Ccr2* were detected with qPCR with different treatments respectively. (**I**) The expression of *Ptgs2, Tlr4* and *Ccr2* were detected with the western blot with different treatments respectively. Data are presented as the mean ± SEM, n = 5, **p < 0.001 versus NC-Anti group, ns p > 0.05 versus MCAO, Mann–Whitney test, ^#^p < 0.05 versus NC-Anti group, t-test. An ELISA examined the protein levels of IL-1β (**J**), TNF-α (**K**), IL-4 (**L**), and IL-10 (**M**) in the infarcted cortex at 24 h after MCAO. Data represent the mean ± SD. n = 5. *p < 0.05, **p < 0.01, and ***p < 0.001 versus sham group; ^##^p < 0.01, and ^###^p < 0.001 versus NC-Anti group, t-test.

To further provide insights into the key genes targeted in anti-inflammatory strategies, we confirmed the mechanism of three hub genes, Ptgs2, Tlr4, and Ccr2. Firstly, we transfected microglia cells with Anti-202-3p and related NC-Anti before the OGD/R insult. Compared to the NC-Anti group, the immuno-fluorescence results showed that the levels of iNOS increased while Arg-1 dropped in the Anti-*202-3p* group (Fig. 9A,B). Secondly, to further confirm the effect of *miR-202-3p* in the MCAO model, we divided mice into 4 groups: sham control group, MCAO only group, MCAO plus NC-Anti group, and MCAO plus Anti*-202-3p* group. The Anti-202-3p was microinjected into the lateral ventricles of the mice, and the knockdown effect of *miR-202-3p* was confirmed (Fig. 10A). After confirming that *miR-202-3p* could be stably knocked down in vivo, MCAO was performed and the neurological function scores of the mice were assessed. The findings revealed that the mice's neurological function was considerably compromised in the Anti-202-3p group and the knockdown of *miR-202-3p* could successfully rescue the brain injury after MCAO/R (Fig. 10B). Interestingly, the Anti-202-3p mice exhibited less cerebral edema (Fig. 10C), and smaller infarct volume demonstrated by TTC staining (Fig. 10D,E). Moreover, the *Ptgs2*/*Tlr4*/*Ccr2* level was upregulated when *miR-202-3p* was knocked down in the MCAO mice model (Fig. 10F–H). The protein levels for *Ptgs2*/*Tlr4*/*Ccr2* were also measured with western blot assays (Fig. 10I), indicating that the *Ptgs2*/*Tlr4*/*Ccr2* level was upregulated when *miR-202-3p* was knocked down in the MCAO mice model. We then explored the effect of *miR-202-3p* knockdown in modulating the levels of inflammatory cytokines. Two pro-inflammatory cytokines—IL-1β and TNF-α (Fig. 10J,K) and two anti-inflammatory cytokines—IL-4 and IL-10 (Fig. 10L,M)—were measured by ELISA. After *miR-202-3p* was knocked down, the levels of TNF-α and IL-1β increased, while the levels of IL-4 and IL-10 decreased. These promising results suggest that *miR-202-3p* regulates the *Ptgs2*/*Tlr4*/*Ccr2* genes in the inflammation response of IS.

## Discussion

Persistent neuroinflammation plays an important role in many pathological processes during the acute phase of IS^13^. However, the changes in gene patterns and the specific mechanisms of inflammation have not been fully clarified^14^. As the treatment window continues to expand, the management of IS increasingly relies on recanalization therapy^15^. Unfortunately, the restoration of blood flow increases oxidative stress in ischemia tissues^16^ and enhances the release of pro-inflammatory cytokines^17^, leading to a series of pathological cascades that result in apoptosis, breakdown of the blood–brain barrier, hemorrhagic transformation, and cerebral edema. A previous study has revealed that neuroinflammatory mechanisms closely associated with oxidative stress contribute significantly to neuronal damage during the acute phase of IS^18^, ultimately exacerbating the extent of neurological deficit and cerebral damage caused by IRI^19^. However, in later stages of cerebral ischemia, neuroinflammatory pathways play an active role in tissue repair and functional recovery^20^. In this study, we identified three genes, *Ptgs2*, *Tlr4,* and *Ccr2* by combining bioinformatics and animal experiments, and found that their expression increased during the acute phase after IRI. Theoretically, these genes are simultaneously regulated by an important down-regulated microRNA, *miR-202-3p*. These findings may shed light on the molecular mechanisms related to neuroinflammation in IS.

In the central nervous system (CNS), microglia are considered a major source of inflammatory mediators^21^. The sudden interruption of blood supply to the brain triggers an immune response, leading to loss of energy and neuronal damage, ultimately activating inflammatory cells^22^. In damaged neurons, the brain is protected from IS by various processes. Gene expression is rapidly altered, and various factors, such as ATP and glutamate, are produced, promoting the activation and migration of nearby microglia^23^. Over-activated microglia are also thought to activate different inflammatory pathways, such as the nuclear factor-κB (NF-κB) pathway, releasing large amounts of proinflammatory mediators such as IL-1β, IL-6, and TNF-α, leading to acute inflammatory reactions. Overproduction of inflammatory cytokines exacerbates neighboring neuronal damage and leads to delayed deterioration of ischemic tissue^24^. Therefore, identifying key regulators that inhibit inflammatory factors produced by microglia and targeting these signaling molecules could prevent neuronal death after IS. Previous studies have shown that miRNAs can prevent excitotoxic injury by inhibiting post-stroke inflammation and oxidative stress in vitro and in vivo^25^. In this study, we found that *miR-202-3p* expression was down-regulated in mice 24 h peri-infarct tissue after IRI. In contrast to previous studies, we proposed three new candidate target genes for *miR-202-3p*. This study confirms that *miR-202-3p* is a promising target for the treatment of cerebral IRI in the acute phase. Furthermore, we linked several genes to this target and explored their mutual regulation during inflammation. Our study may provide new insights into in-depth research on inflammatory mechanisms after IS.

*TLR4* is a potential target gene of *miR-202-3p*. We observed significant upregulation of *TLR4* in the MCAO model. Toll-like receptors are pattern recognition receptors that recognize pathogen-associated molecular patterns and endogenous danger-associated molecular patterns^26^. Among TLRs present in the mammalian CNS, *TLR4* is mainly expressed in microglia and mediates neuroinflammatory diseases^27^. Previous studies have shown that the TLR4 pathway plays a role in microglial polarization in neurodegenerative diseases such as Alzheimer's disease^28^ and Parkinson's disease^29^, as well as CNS trauma such as traumatic brain injury^30^ and intracerebral hemorrhage^31^. Inhibition of *TLR4* may have a positive effect on tissue homeostasis and reduce ischemic damage in the myocardium and spinal cord^32^. An in vitro study has demonstrated that the activated TLR4/NF-κB pathway microglial polarization to the M1 phenotype after IS^33^. Our findings confirm that *TLR4* is an important factor in the regulation of the inflammatory response in the acute phase of IS and may be a potential therapeutic target.

*Ptgs2* is another candidate gene targeting *miR-202-3p*, a key response gene associated with homeostasis in the regulatory inflammatory response^34^. Several studies have investigated the relationship between *Ptgs2* gene polymorphisms and IS^35^. Consistent with our study, *Ptgs2* expression is significantly increased in stroke with inflammatory cell infiltration, and related studies have reported a correlation between Ptgs2 and IS^36^. Previous studies have suggested that *Ptgs2* may be associated with tumor aggressiveness and is enriched in a variety of primary endometrial tumors^37^. During cerebral ischemia, when inflammatory cells infiltrate the injured brain, *Ptgs2* is found to be upregulated^38^. Furthermore, transient cerebral ischemia has been shown to induce marked apoptosis and inflammatory responses such as NF-κB activation^39^. Hence, *Ptgs2* is a protein closely associated with inflammatory processes and has potential applications.

In our study, we confirmed *Ccr2* as the third gene that may interact with *miR-202-3p*, which is also upregulated in the ischemic penumbra of mice. Chemokine receptor *Ccr2* is associated with the release and migration of monocytes to the ischemic brain, when activated by its ligand, Ccl2^40^. *Ccr2*/*Ccl2* is known to function in many CNS disorders including ischemic encephalopathy^41^. However, there is no definitive conclusion regarding the role of *Ccr2*-dependent monocytes/macrophages in acute brain injury after IS. Some studies show that knockdown or pharmacological inhibition of *Ccr2* suppresses acute brain injury^42^, while others show that selective *Ccr2* antagonist causes greater damage in ischemic rat models^43^. Hence, the exact role of *Ccr2*-dependent MoDMs remains unclear, and how they affect the recovery of brain function after IS remains largely unknown. Nevertheless, our study confirms that *Ccr2* is significantly elevated during the acute phase of IS and is modulated by *miR-202-3p*, suggesting that it may be a potential therapeutic target for IS.

Current studies on microglia phenotype have mainly focused on the process of miRNA-reducing pro-inflammatory response, with less emphasis on the anti-inflammatory effects of the M2 phenotype. Therefore, shifting the research focus to miRNAs related to the neuro-inflammatory networks would be a novel idea to study the regulation of neuroinflammation. Moreover, a more comprehensive understanding of the impact of miRNAs on neuroinflammation involving microglia may lead to the development of new therapeutic targets. For instance, utilizing miRNA delivery systems to regulate the expression of key proteins associated with specific diseases could be a promising approach^44^. Current miRNA-based therapies compromise miRNA inhibitors and miRNA mimetics^45^. Several immunomodulators have been shown to modulate microglial neuroinflammation by inhibiting miRNA function^46^. In this study, we used bioinformatics to identify and validate the differential expression of three target genes of *miR-202-3p*, *Ptgs2*, *Tlr4,* and *Ccr2*, in the cerebral ischemia penumbra of mice. This promising new approach can help to investigate the inflammatory mechanisms of IS.

The interrelationship between the aforementioned genes and their dual role in the constantly changing inflammatory network of ischemia naturally attracted the attention of scholars. Thus, we established an interaction network between *Ptgs2*, *Tlr4*, and *Ccr2* and comprehensively analyzed their variation within the acute phase of IRI. In our study, we found that the selected three genes were significantly increased after ischemia–reperfusion, providing evidence that changes in their levels could be a potential therapeutic target. In addition, *MiR-202-3p* deduced from the gene examined in this study, was found to regulate three of the 11 key genes through bioinformatics analysis, suggesting that it plays a central role in the regulation of inflammation in the acute phase of IS. Furthermore, our study showed that *miR-202-3p* was significantly decreased and *Ptgs2*, *Tlr4*, and *Ccr2* were increased 24 h after IRI in vivo*.*

More attention should be given to how changes in *Ptgs2*, *Tlr4*, and *Ccr2* levels affect their function during acute IS and their interactions with *miR-202-3p*. Unfortunately, our research has some limitations. First, the inflammatory response in IS is dynamic and the results may be influenced by the timing and severity of the stroke animal model, as well as the samples collected. Second, the data analyzed were obtained from online databases and rodents. For further studies, more human tissue samples and data are required to validate our results. Additionally, the association of *miR-202-3p* with *Ptgs2*, *Tlr4*, and *Ccr2* was not directly verified, and their potential correlation mechanism in ischemic brain injury needs further investigation. Finally, using animal models to elucidate the molecular mechanisms of human disease has the drawback of difficulty in obtaining critical brain tissue from humans with IS. Therefore, studies with surgical specimens or donor brain tissue from deceased stroke patients are needed to elucidate possible mechanisms.

## Conclusion

Our study suggests that the differentially expressed genes *Ptgs2*, *Tlr4,* and *Ccr2* play a critical role in regulating inflammation in IS and are negatively regulated by *miR-202-3p*. These findings provide novel insights into the complex pathophysiological mechanisms of neuroinflammatory after IS.

## Materials and methods

### Acquisition of RNA data

The microarray expression datasets GSE58720 and GSE202659 were obtained from the GEO database https://www.ncbi.nlm.nih.gov/geo). We selected 6 samples, including 3 SHAM models and 3 MCAO/R models from GSE58720. Similarly, we selected 6 samples, including 3 SHAM models and 3 MCAO/R models, from GSE202659.

### Differential expression genes

The GEO2R online analysis tool (https://www.ncbi.nlm.nih.gov/geo/geo2r/) was used to explore the DEGs between SHAM and MCAO/R samples, and the adjusted P-value and |logFC| were calculated. Differentially expressed genes (DEGs) were defined as those with an adjusted P-value. The intersecting part was identified with the Venn diagram web tool (bioinformatics.psb.ugent.be/webtools/Venn/).

### Gene set enrichment analysis (GSEA)

Gene Set Enrichment Analysis (GSEA) sequenced the genes based on the differential expression levels in the two groups and then detected whether the preset gene set was enriched at the top or bottom of the sequencing table. The genetic information of the SHAM and MCAO/R samples was uploaded to R software and analyzed using the limma package.

### DAVID analysis

To explore the pathways and functions of the 168 overlapping genes, we performed Gene Ontology (GO) and Kyoto Encyclopedia of Genes and Genomes (KEGG) pathways analyses with the DAVID 6.8 online tools (https://david.ncifcrf.gov/).

### Metascape functional annotation and pathway analysis

We uploaded 170 overlapping genes to the Metascape online analysis tools (https://metascape.org/) to perform GO analysis, including biological process (BP), molecular function (MF), cellular component (CC), and KEGG pathway enrichment. We used the following parameters: Min Overlap = 3, P-value Cutoff = 0.01, Min Enrichment = 1.5.

### Construction of protein–protein interaction (PPI) network

We inputted the DEGs into the Metascape (https://metascape.org/) and STRING (https://www.string-db.org/) online databases to obtain the protein network interaction diagram. The edges indicate both functional and physical protein associations. The line color indicates the type of interaction evidence, and the minimum required interaction score was set at medium confidence (0.4). To visualize the PPI network, we used Cytoscape software (version 3.8.2). We uploaded 168 overlapping genes to Cytoscape software to visualize the interaction network of the biological process with the Cytoscape ClueGo plug-in.

### Construction of miRNA-mRNA network

The target genes from DEGs were inputted into the miRWalk online program (http://mirwalk.umm.uni-heidelberg.de/) to predict the key miRNAs. Finally, the relevant results were applied to Cytoscape software to construct the mRNA–miRNA correlation network. The selection criteria were set as seed match > 7 mer-1A, the context++ score percentile > 90, and the target gene binding region was 3′UTR.

### MCAO model

The MCAO surgery was performed as previously described^47^. Briefly, mice were given 2% isoflurane in oxygen for 3 min, followed by 1.0–1.5% isoflurane in 70% N_2_O and 30% O_2_, and anesthesia was maintained by a small-animal anesthesia system. A midline neck incision was made to separate the left external carotid artery (ECA) and common carotid artery (CCA). After temporarily blocking the common and internal carotid arteries (ICA), a silicone rubber monofilament was inserted through the arteriotomy into the ECA and slowly advanced to the beginning of the middle cerebral artery (MCA) through the left ICA. After 1 h of occlusion, the monofilament was removed and the ECA was permanently ligated. For the sham group, only the monofilament was inserted into the rats to block the MCA, and then the monofilament was removed and blood flow was rapidly restored.

### Quantitative real-time PCR

To extract RNA from tissues, TRIzol reagent was used. For qRT-PCR, the SYBR Green system and specific primers were utilized. The relative Ct method was used to compare the outcomes between the control and experimental groups, with β-actin serving as an internal reference. The primers used for real-time quantitative PCR (RT-qPCR) are shown in Supplementary Table 1.

### Western blot analysis

Cells or tissues were washed with cold PBS and lysed with RIPA buffer containing PMSF and phosphorylated protease inhibitors. The lysates were then centrifuged at 12,000×*g* for 10 min at 4 °C, and the protein level in the supernatant was determined using a BCA protein assay. Next, the lysates were mixed with 5% SDS-PAGE and boiled for 10 min at 98 °C. The total proteins were loaded onto an SDS-PAGE gel and electrically transferred to a 0.22 μM PVDF membrane. The membrane was blocked with 5% skim milk in Tris-buffered saline. The primary antibody was incubated with the membrane overnight in a ratio of 1:1000 at 4 °C. Primary antibodies are listed in Supplementary Table 2. The membrane was then incubated with Horseradish peroxidase secondary antibodies in a ratio of 1:2000 for 2 h at 25 °C. Finally, a fixed western chemiluminescent HRP substrate (Micropores) was used to visualize the protein bands.

### Oxygen–glucose deprivation (OGD)

Microglia cells were obtained from the Cell Bank Type Culture Collection of the Chinese Academy of Sciences and cultured in ECM medium containing 10% fetal bovine serum (FBS), 100 μg/ mL penicillin, and 100 μg/ mL streptomycin at 37 °C in 5% CO_2_. The cells were incubated at 37 °C with 95% N_2_, and 5% CO_2_ for 24 h before transfection with DMEM without glucose, after which the cells were transferred to a six-well plate. The cells were then cultured under normoxic conditions for 24 h.

### Cell transfection

*MiR-202-3p* inhibitors (Anti-*202-3p*) and their corresponding control (NC-Anti) were purchased from Invitrogen. Microglia cells were transfected with either Anti-*202-3p* or NC-Anti using Lipofectamine 2000 according to the manufacturer’s instructions. After transfection with NC-Anti or Anti-*202-3p* for 24 h, the microglia cells were exposed to either normoxia or OGD for 4 h.

### Immune-fluorescence assay

The cells were fixed in 4% paraformaldehyde for 30 min, followed by permeabilization with 0.1% Triton X-100 for 10 min. Subsequently, the cells were blocked with 1% bovine serum albumin for 1 h and incubated overnight with primary antibodies specified in Supplementary Table 2. Finally, the cells were incubated with fluor-labeled secondary antibodies, and the nuclei were stained with DAPI. The images were captured using either an Olympus BX51 microscope or an Olympus FV1200 confocal microscope.

### Animals

Four-week-old C57BL/6J male mice were purchased from Shulaibao Biotechnology Co., Ltd. They were housed under constant humidity and temperature and allowed free access to food and water. All mice experiments were conducted on a specific pathogen-free platform at the Experimental Animal Center of the Renmin Hospital, Wuhan University. The mice were randomly allocated into different groups and were matched for body weight. All animal experiments were approved by the Ethics Committee of Renmin Hospital of Wuhan university (Approval Number: WDRM-20200301C) and performed in line with the AAALAC and the ARRIVE guidelines.

### Intracerebroventricular administration

Anti-*202-3p* or NC-Anti were transfected into mice brains as described previously^48^. Briefly, the mice were deeply anesthetized and the plasmid premixed with transfection reagent was microinjected into the left lateral ventricle at a rate of 0.2 µL/min. The microsyringe was left in place for 10 min after the intracerebroventricular injection. In the Sham group, mice were subjected only to a burr hole and administered an equal amount of saline through injection.

### Neurobehavioral assessment

The modified neurological Severity score (mNSS) was used to assess the neurological impairment of the groups mentioned above (n = 5 in each group) at 24 h post MCAO. Neurological function was rated on a scale of 0 to 18 (normal is 0; with a maximum loss score 18 points). A score of 1 on the test indicates either the inability to perform the test or a lack of test reflexes. Therefore, a higher score indicates a more severe injury.

### TTC staining

Mice were euthanized in a humanely within 24 h after MCAO. The euthanasia was performed by a rapid intravenous injection of sodium pentobarbital at 75 mg per kg of body weight. Brain tissue was excised and collected. It was immediately divided into four coronal sections. They were then incubated for 30 min with the 2% TTC solution at 37 °C before it was transferred to a 4% paraformaldehyde solution and fixed for one day. Using Image J software, we measured the extent of infarction in each section on each captured digital images.

### Brain water content analysis

The brains were extracted within 24 h after MCAO and wet weights of the right and left hemispheres were calculated. To determine dry weight, the brains were boiled for 48 h at 75 °C. Subsequently, the water content of the brain was calculated as [(wet weight − dry weight)/wet weight] × 100%.

### ELISA

At 24 h after MCAO, mice were anesthetized and underwent transcardial perfusion with phosphate-buffered saline (PBS, pH 7.4, 4 °C). Brain infarcts zone were immediately collected, homogenized with 200 mg/mL of 0.9% normal saline, and centrifuged at 12,000 rpm. The supernatant was collected and kept at − 80 °C. The quantities of interleukin 10 (IL-10), tumor necrosis factor alpha (TNF-α), interleukin 4 (IL-4) and interleukin 1β (IL-1β) in brain tissue lysates were measured using commercial ELISA kits. The ultimate concentration of cytokines was calculated from the absorbance standard curve following the manufacturer’s protocol.

### Statistical analysis

Statistical analysis was conducted using GraphPad Prism 8.0. All data were expressed as mean ± SD. One-way ANOVA and t-test were performed to compare differences between groups. Each experiment was repeated three times. P < 0.05 was considered statistically significant.

### Supplementary Information

Supplementary Information 1.

Supplementary Information 2.

Supplementary Information 3.

Supplementary Information 4.

## Data Availability

The raw data supporting the conclusions of this article will be made available by the authors, without undue reservation.
