# Supplementary figures and images for: Essential genes Ptgs2, Tlr4, and Ccr2 regulate neuro-inflammation during the acute phase of cerebral ischemic in mice
Source: Sci Rep. 2023 Aug 10;13:13021. doi: 10.1038/s41598-023-40255-w (PMC10415315; doi:10.1038/s41598-023-40255-w)

Tlr4:

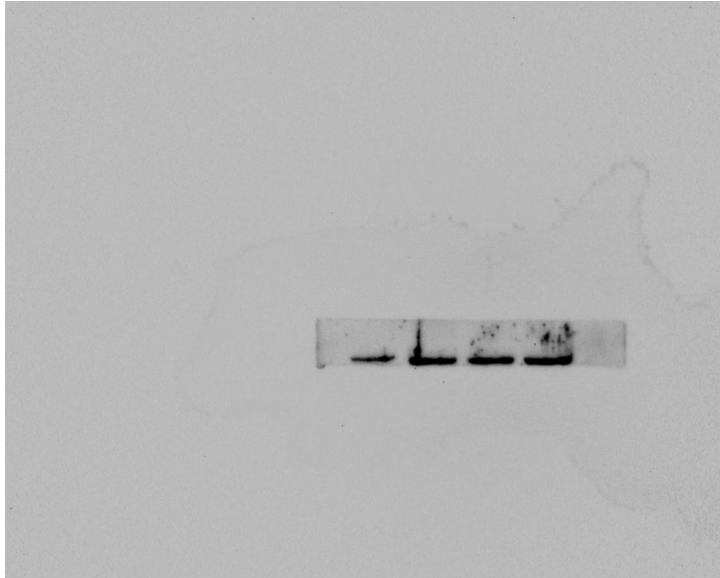

Ptgs2:

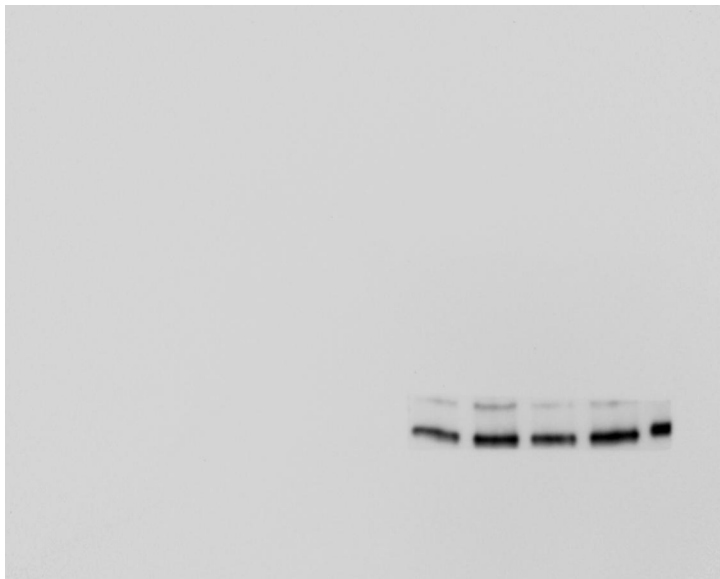

Ccr2:

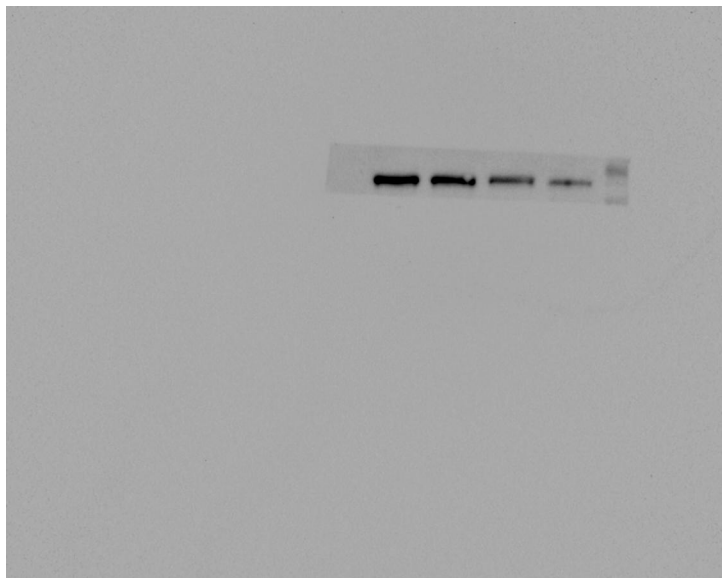

GAPDH:

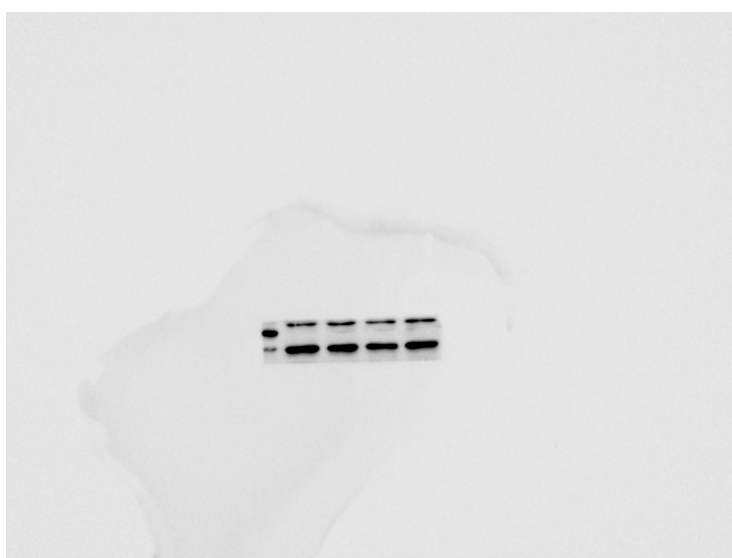

Supplement: Supplementary file 1 — Supplementary Information 1. [file 41598_2023_40255_MOESM1_ESM.pdf]

Ptgs2:

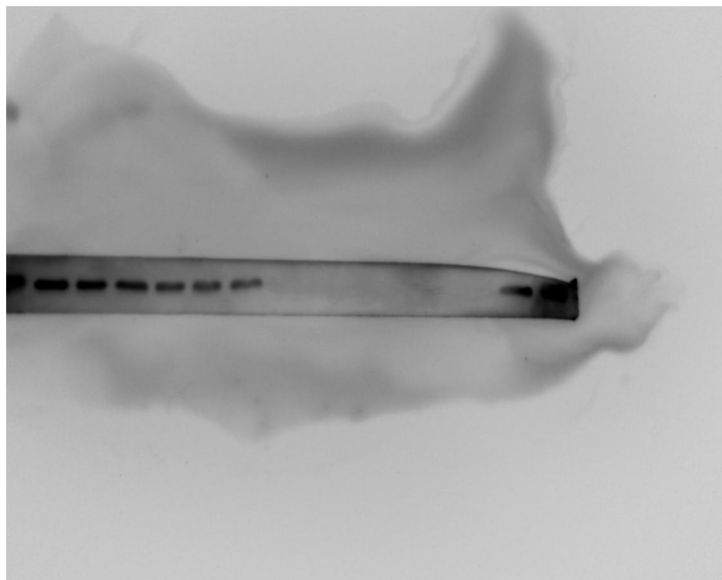

Ccr2:

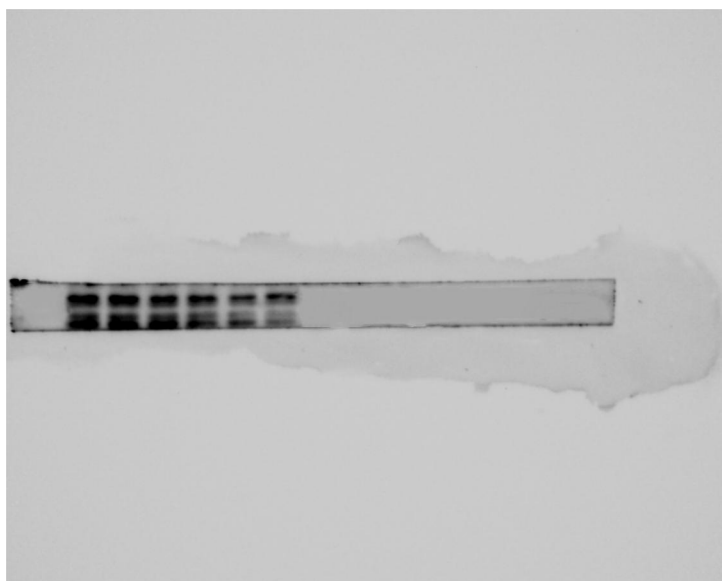

Tlr4:

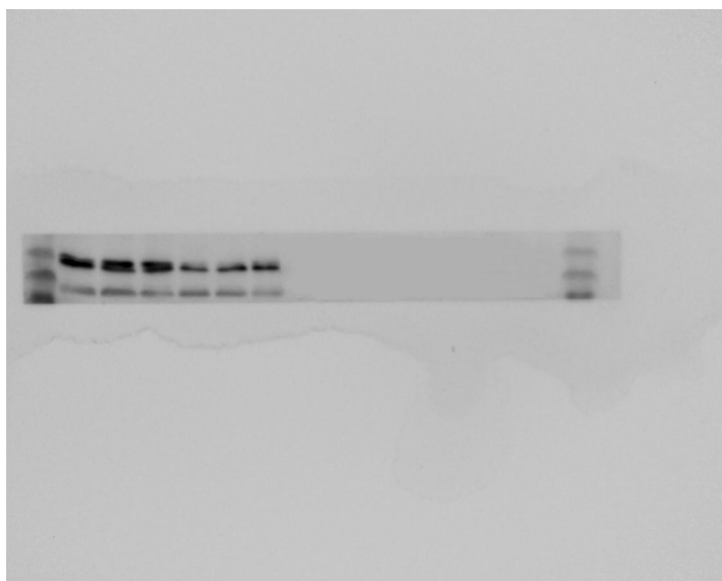

GAPDH:

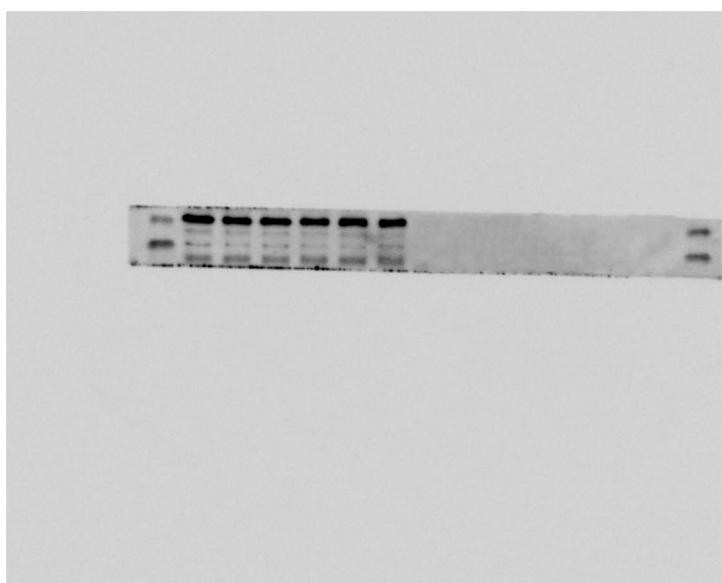

Supplement: Supplementary file 2 — Supplementary Information 2. [file 41598_2023_40255_MOESM2_ESM.pdf]
