# Supplementary material for: Essential genes Ptgs2, Tlr4, and Ccr2 regulate neuro-inflammation during the acute phase of cerebral ischemic in mice
Source: Sci Rep. 2023 Aug 10;13:13021. doi: 10.1038/s41598-023-40255-w (PMC10415315; doi:10.1038/s41598-023-40255-w)
Supplement: Supplementary file 3 — Supplementary Information 3. [file 41598_2023_40255_MOESM3_ESM.doc]

**Table S1** PCR primers sequences

| mRNA | Forward sequence | Reverse sequence |
| --- | --- | --- |
| miR-202-3p | 5'-TTCCTCCTGTGCCTGATGATT-3' | 5'-AAACTGATGCGTGAAGTGCTG-3' |
| Ptgs2 | 5'-TTCCTCCTGTGCCTGATGATT-3' | 5'-AAACTGATGCGTGAAGTGCTG-3' |
| Tlr4 | 5'-AGTTTCCTGCAATGGATCAAGG-3' | 5'-CTGCTTATCTGAAGGTGTTGCAC-3' |
| Ccr2 | 5'-GGCCAGCAAATTACCTGTGTG-3' | 5'-AGGCGGACATCCTGAACCT-3' |
| GAPDH | 5'-GGCTGTTGTCATA CT TCTCATGG-3' | 5'-GGAGCG AGATCCCTCCAAAAT-3' |

Abbreviations: Ptgs2, Prostaglandin endoperoxide synthase 2; Tlr4，Toll-like receptor 4; Ccr2, CC chemokine receptor 2; GAPDH as the internal control.
