# Supplementary material for: Essential genes Ptgs2, Tlr4, and Ccr2 regulate neuro-inflammation during the acute phase of cerebral ischemic in mice
Source: Sci Rep. 2023 Aug 10;13:13021. doi: 10.1038/s41598-023-40255-w (PMC10415315; doi:10.1038/s41598-023-40255-w)
Supplement: Supplementary file 4 — Supplementary Information 4. [file 41598_2023_40255_MOESM4_ESM.doc]

**Table S2** Antibodies

| Target | Label | Species | Catalog | Concentration | Company |
| --- | --- | --- | --- | --- | --- |
| anti-Ptgs2 |  | goat | ab5076 | 1:400 | abcam |
| anti-Tlr4 |  | rabbit | sc-71102 | 1:500 | Santa Cruz Biotechnology |
| anti-Ccr2 |  | rabbit | sc-13515 | 1:500 | Santa Cruz Biotechnology |
| anti-Arg-1 |  | rabbit | #12282 |  | Cell Signaling |
| anti-iNOS |  | rabbit | 18985-1-AP | 1:1000 | Proteintech |
| anti-gapdh |  | rabbit | A5441 | 1:1000 | Sigma-Aldrich |
| anti-goat | AF647 | donkey | Cat. 705-605-003 | 1:500 | Jackson Immuno-Research |
| anti-goat | biotin | donkey | B7024 | 1:250 | Sigma |
| anti-rabbit | biotin | donkey | 150073 | 1:250 | Abcam |
